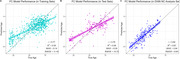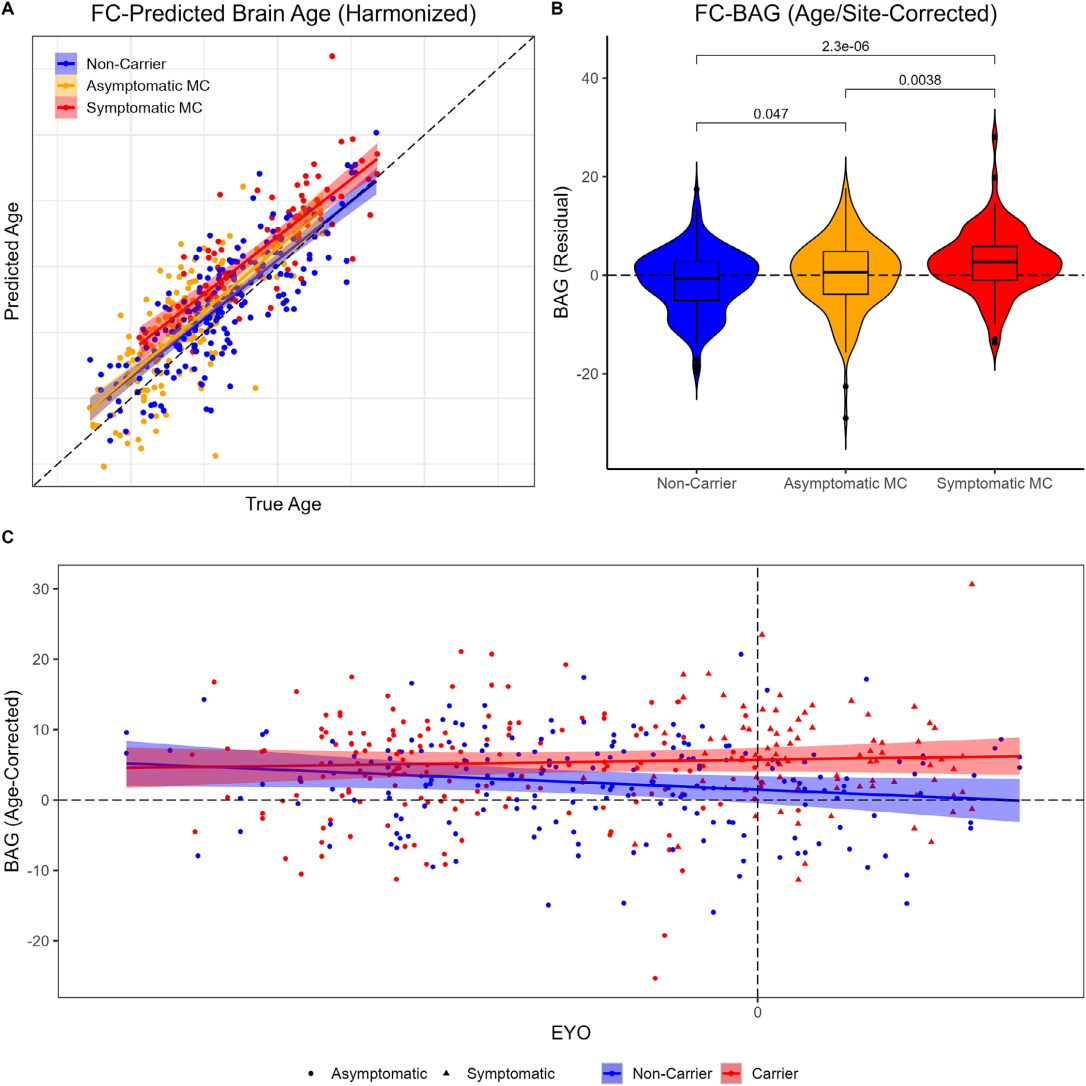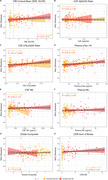# Evaluating brain age using functional connectivity in autosomal dominant Alzheimer disease

**DOI:** 10.1002/alz.084990

**Published:** 2025-01-09

**Authors:** Kiana Angela Macharia, Brian A. Gordon, Julie K. Wisch, Nicholas V Metcalf, June Roman, Tammie L.S. Benzinger, Carlos Cruchaga, Alison M. Goate, Jason J. Hassenstab, Laura Ibanez, Celeste M. Karch, Jorge J. Llibre‐Guerra, John C. Morris, Richard J. Perrin, Alan E. Renton, Charlene B Supnet, Chengjie Xiong, Dominantly Inherited Alzheimer Network (DIAN), Randall J. Bateman, Eric McDade, Beau Ances, Peter R Millar

**Affiliations:** ^1^ Washington University in St. Louis School of Medicine, St. Louis, MO USA; ^2^ Washington University in St. Louis, St. Louis, MO USA; ^3^ Washington University in St. Louis, School of Medicine, St. Louis, MO USA; ^4^ Icahn School of Medicine at Mount Sinai, New York, NY USA; ^5^ Department of Psychiatry, Washington University in St. Louis School of Medicine, St. Louis, MO USA

## Abstract

**Background:**

Brain‐predicted age estimates are used to quantify an individual's brain age compared to a normative trajectory. We have recently shown that brain age from structural MRI is elevated in autosomal dominant Alzheimer disease (ADAD), a unique sample that allows the study of AD progression independently of age‐related confounds. Resting‐state functional connectivity (FC) may capture a biphasic response to sporadic AD, and thus may complement structural measures of brain aging in ADAD.

**Method:**

We trained a Gaussian Process Regression (GPR) model to predict age from FC in 271 regions of interest in 779 cognitively unimpaired, amyloid‐negative, non‐ADAD participants. We applied the model in a cohort of 256 ADAD mutation carriers (MCs) and 200 familial non‐carrier controls (NCs) from the Dominantly Inherited Alzheimer Network (DIAN). We then assessed whether the brain age gap (FC‐BAG; difference between predicted and actual age) varied based on mutation status, cognitive status, or estimated years until symptom onset (EYO). We also explored its associations with markers of amyloid (PiB PET, CSF amyloid‐β‐42/40), phosphorylated tau (CSF and plasma pTau‐181), neurodegeneration (CSF and plasma neurofilament light (NfL)), and cognition (global neuropsychological composite and CDR®‐sum of boxes).

**Result:**

The model accurately predicted age in the training set (r = 0.77), validation set (r = 0.76), and DIAN NCs (r = 0.82), Figure 1. FC‐BAG was elevated in symptomatic MCs (p < .001), but only marginal elevation was observed in asymptomatic MCs (p = 0.047), Figure 2. In symptomatic MCs, FC‐BAG weakly associated with pTau‐181 in CSF (r = 0.34, p = 0.0046) and plasma (r = 0.26, p = 0.02), but no other associations with AD biomarkers or cognition were observed, Figure 3.

**Conclusion:**

FC‐BAG was elevated in symptomatic ADAD, consistent with recent demonstrations in sporadic AD. However, we did not detect a predicted biphasic response to presymptomatic ADAD pathology. This inconsistency may be driven by differences between AD forms or by low reliability of FC, which may result in low statistical power and inconsistent brain‐behavior relationships. FC‐based BAG offers unique benefits but ultimately does not function as a reliable model for observing advanced aging in preclinical ADAD.